# Optimal Bands Combination Selection for Extracting Garlic Planting Area with Multi-Temporal Sentinel-2 Imagery

**DOI:** 10.3390/s21165556

**Published:** 2021-08-18

**Authors:** Shuang Wu, Han Lu, Hongliang Guan, Yong Chen, Danyu Qiao, Lei Deng

**Affiliations:** 1 College of Resource Environment and Tourism, Capital Normal University, Beijing 100048, China; 2200902139@cnu.edu.cn (S.W.); 2200901010@cnu.edu.cn (H.L.); hlguan@cnu.edu.cn (H.G.); 2190902133@cnu.edu.cn (Y.C.); 2190902135@cnu.edu.cn (D.Q.); 2 Engineering Research Center of Spatial Information Technology, Ministry of Education, Capital Normal University, Beijing 100048, China; 3 Beijing Laboratory of Water Resources Security, Capital Normal University, Beijing 100048, China

**Keywords:** multi-temporal, garlic, band combination, planting area, Sentinel-2

## Abstract

Garlic is one of the main economic crops in China. Accurate and timely extraction of the garlic planting area is critical for adjusting the agricultural planting structure and implementing rural policy actions. Crop extraction methods based on remote sensing usually use spectral–temporal features. Still, for garlic extraction, most methods simply combine all multi-temporal images. There has been a lack of research on each band’s function in each multi-temporal image and optimal bands combination. To systematically explore the potential of the multi-temporal method for garlic extraction, we obtained a series of Sentinel-2 images in the whole garlic growth cycle. The importance of each band in all these images was ranked by the random forest (RF) method. According to the importance score of each band, eight different multi-temporal combination schemes were designed. The RF classifier was employed to extract garlic planting area, and the accuracy of the eight schemes was compared. The results show that (1) the Scheme VI (the top 39 bands in importance score) achieved the best accuracy of 98.65%, which is 6% higher than the optimal mono-temporal (February, wintering period) result, and (2) the red-edge band and the shortwave-infrared band played an essential role in accurate garlic extraction. This study gives inspiration in selecting the remotely sensed data source, the band, and phenology for accurately extracting garlic planting area, which could be transferred to other sites with larger areas and similar agriculture structures.

## 1. Introduction

Garlic is a cash crop with a high yield. Under the current conditions of low planting benefit, expanding the garlic planting area plays a critical role in improving the overall use of agricultural production and increasing farmers’ income [1,2,3]. In 2019, China produced 23.306 million tons of garlic, accounting for 75.9% of the global output and ranking first in the world. However, because of the irregular distribution of garlic planting areas and the high degree of fragmentation, traditional garlic distribution information is based primarily on field sampling surveys and step-by-step statistics, which are time-consuming, laborious, subjective, and have a time lag [4]. As a result, the distribution of garlic cannot be expressed spatially.

Remote sensing technology has been continuously developed over the recent decades, and it is now widely used in crop extraction [5,6,7]. The use of satellite remote sensing technology in crop extraction can significantly reduce workload, improve efficiency, and ensure data objectivity. In addition, it can obtain crop planting area and specific spatial distribution simultaneously, which is helpful for monitoring crop planting in different locations [8,9,10]. Crop extraction and surface features classification are commonly performed using mono-temporal satellite images with medium and high spatial resolutions [11,12,13,14,15,16]. Some researchers, for example, use mono-temporal Landsat images to extract crop planting areas such as wheat and rice with high accuracy [14,16]. Still, the pixels at the intersection of the extracted crops and other surface features frequently have similar spectral characteristics, leading to confusion. Furthermore, some researchers use mono-temporal high-resolution GeoEye-1 [15] images to extract vegetation and IKONOS [11] satellite images to extract urban debris areas, which has achieved good results. However, there are still some other surface features that are not wholly distinguished.

Despite some progress in crop recognition and extraction using mono-temporal images, it is challenging to distinguish crop types accurately using only mono-temporal remote sensing images. Due to physical and chemical characteristics of crops, such as pigment and water content, they have a certain similarity during the growth period. It leads to the high similarity of spectral characteristics of different crops, making them susceptible to the phenomenon of different surface features with the same spectrum, or different spectrum with the same surface feature. As a result, the use of multi-temporal remote sensing image data is critical [17]. Sentinel-2, the European Space Agency multi-spectral satellite, can provide a wealth of spectral and temporal information for crop classification and extraction [18,19]. One satellite (Sentinel-2A or Sentinel-2B) has a 10-day revisit period, and the two satellites complement each other, reducing the time resolution to 5 days and greatly improving the level of land monitoring. Sentinel-2 has multi-spectral bands ranging in the visible-red edge-near infrared-shortwave infrared spectrum. It is the only dataset with three bands in the red-edge range, making it ideal for monitoring vegetation health [18,20,21,22,23,24]. It has also made significant advances in remote sensing monitoring of large-scale food crops (such as winter wheat [14,25], corn [26], and rice [26,27]).

It should be noted that although using multi-temporal data can improve crop extraction accuracy, not all images from all periods and/or all bands are effective in improving accuracy [28]. On the contrary, the increase in the number of images may bring a decrease in accuracy, which is known as Hughes effect [29,30]. Pal et al. [29] discovered that increasing the number of hyperspectral bands used to classify land use cover reduced classification performance to some extent. Meng et al. [31] found that as the number of Sentinel-2 images used in classification increases, classification performance decreases, affecting crop extraction. In addition, the previous studies used all multi-temporal images of the crop’s whole growth cycle directly for extraction. Few studies systematically focused on garlic, such as which period or combination of periods was best for garlic extraction and which band was the best for improving accuracy. As a result, it is necessary to improve garlic extraction accuracy by systematically combining bands from images in different periods.

This study aimed to find the optimal combination of the multi-temporal Sentinel-2 imagery for extracting garlic planting area accurately. Therefore, we used the Mean Decrease Gini [32] in the RF method to rank the importance of all bands in the whole growth cycle of garlic. According to the importance score of each band, we designed eight different multi-temporal schemes. Then, through visual interpretation and comparison with Google Earth images and the area data from official statistics, we evaluated the accuracy of the eight different schemes and compared them with the mono-temporal schemes.

## 2. Study Area and Data

### 2.1. Study Area

**Figure 1:**
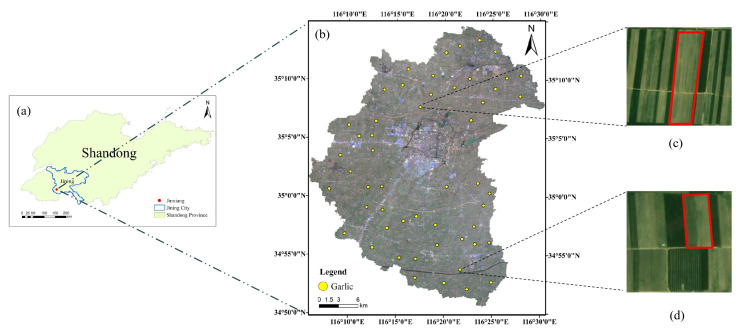
(**a**) The location of the study area; (**b**) the spatial distribution of garlic sample data on the satellite image from 9 February 2020 (true-color composite of the blue, green, and red bands of Sentinel-2 image); (**c**,**d**) the garlic sample data on the Google Earth satellite images.

Jinxiang County (34°50′–35°15′ N, 116°5′–116°30′ E) was chosen as the study area in this study. The study area is southwest of Jining City, Shandong Province, and is the main garlic production area of the Huabei Plain (Figure 1). The study area is not only the dominant garlic cultivation and production area, but it is also an important garlic export base. In China, it is known as the “Hometown of Chinese Garlic”. Jinxiang County covers an area of approximately 88,600 ha, 70% of which is cultivated land. The climate in the region is temperate continental monsoon, with an average annual temperature of about 14 °C and average precipitation of about 694 mm. Garlic is primarily distributed in the northeast and western regions of the study area because the soil is fertile and the cultivated land is concentrated, making it suitable for garlic growth. The planting area of garlic in the central part is less. In the southeast and northwest garlic is planted scattered because garlic is not the main crop.

Garlic in the study area is autumn sowing garlic, which is sown at the end of September or early October and harvested from May to June of the following year, with eight stages: sowing, germination, seedling, wintering, reviving, bolting, bulb expanding, and harvest, as shown in Table 1. In addition, garlic and winter wheat are in the same growing season, and other crops are rarely planted in this growing season.

### 2.2. Remote Sensing Data

According to the principle of low cloud cover (less than 10%) and as consistent an image time interval as possible, and taking into account the garlic growth period and phenological characteristics, 11 high-quality Sentinel-2 multi-spectral Level-2A (L2A) images covering the entire garlic growth period from September 2019 to July 2020 were selected (Table 1). Sentinel Scientific Data Hub (https://scihub.copernicus.eu/; accessed on 15 January 2021) was used to obtain these images. Sentinel-2 L2A data is reflectance at the bottom of the atmosphere (BOA) after radiation calibration and atmospheric correction. To avoid errors due to water absorption, the three 60 m atmospheric bands 1 (coastal aerosol), 9 (water vapor), and 10 (Cirrus) were removed from the analysis, leaving only the bands most commonly used in land applications. To ensure that all images have the same spatial resolution, the Bilinear Interpolation method was used for all bands of each image. The reflectance 20 m bands were resampled to 10 m in SNAP (ESA Sentinel Application Platform v2.0.2, http://step.esa.int; accessed on 15 January 2021, Brockmann Consult, Skywatch, Sensar, and C-S), and the spectral information was not significantly changed compared to the input image.

### 2.3. Reference Data

Two types of reference data were used to validate the accuracy of garlic extraction. The first reference data is Google’s high spatial resolution imagery. False color was applied to Sentinel-2 images (bands 11, 8 and 4) [14], registered with Google high spatial resolution image to find the location of garlic, and then samples (including training and validation samples) were selected through visual interpretation. Considering the images of all dates except September (sowing period), June and July (harvest period), 138 garlic sample areas were selected, covering 5733 pixels for each date of Sentinel-2 images (Table 2). The samples of non-garlic were also selected (hereinafter referred to as Others). Most of them are winter wheat, and a small number of them are water, buildings, bare land, etc. As shown in Table 2, for each date of images, 5055 pixels from the “others” class were used as training samples and 2400 pixels as validation samples. Furthermore, two typical quadrats of 1 × 1 km^2^ (garlic planting dense area and sparse area) were selected to verify surface features area extraction accuracy further. The boundaries of surface features within each quadrate were manually plotted based on Google Earth imagery, and its spatial resolution was 1 m. The attributes of surface features were identified from Google’s high spatial resolution imagery, and these surface features were deemed to be ground-truth data.

The second reference data is official statistical data on the garlic planting area (http://www.jinxiang.gov.cn/; accessed on 26 February 2021), which was used as a reference for the total amount control, and the total area accuracy of the garlic extraction was then verified. In addition, administrative vector data from Jinxiang County, and garlic phenology information for Jinxiang County in 2019 were used in this study as auxiliary data.

## 3. Method

**Figure 2:**
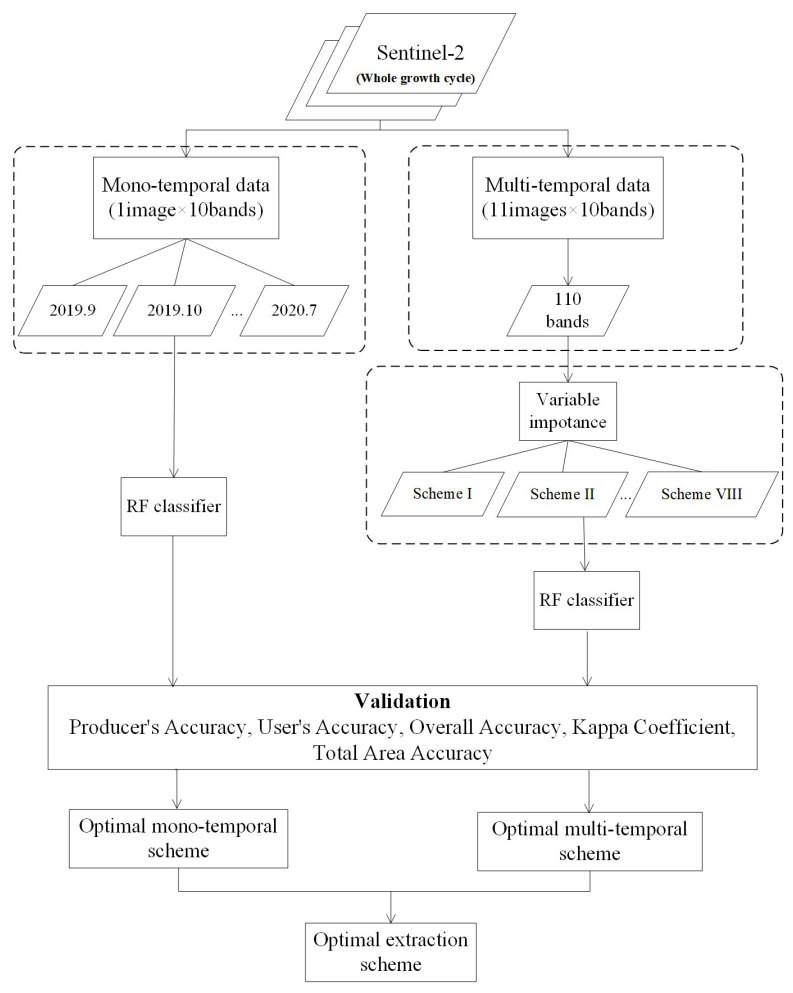
The workflow of optimum extraction scheme.

Figure 2 depicts the study workflow. Based on Sentinel-2 images from the entire growth period of garlic, we developed mono-temporal and multi-temporal garlic extraction schemes and the accuracy of the results was evaluated and analyzed.

First, we divided the Sentinel-2 images of the entire garlic growth cycle into mono-temporal and multi-temporal data sets. The mono-temporal data set represents each month’s image, while the acquisition of multi-temporal data set is to superimpose the bands of all images to generate 110 bands.

Secondly, the RF classifier was used to classify mono-temporal data sets, images of each month were classified into two groups (garlic and others), and the garlic planting area was extracted from them. For 110 bands of multi-temporal data set, to rank the importance for each of the 110 bands, the Mean Decrease Gini [32,33,34] was computed. It is the RF’s meaningful metric about the importance of each variable and widely used in variable selection and importance evaluation in remote sensing [32,34]. The Mean Decrease Gini has a high level of stability because the estimation is unbiased when the variables are continuous and uncorrelated, and it has a high level of accuracy when the signal-to-noise ratio is low. The Mean Decrease Gini is the mean value of a variable’s total decrease in node impurity, weighted by the proportion of samples reaching that node in each individual decision tree in the ensemble. It effectively measures how important a variable is for estimating the value of the target variable across all of the trees that make up the ensemble. A higher Mean Decrease Gini indicates higher variable importance. The Scikit-Learn library and Python 3.9 programming language were used to compute the Mean Decrease Gini. Then, according to the importance scores of 110 bands, we set the step length and designed different schemes by accumulating participating bands. Similarly, the multi-temporal schemes were classified into two groups (garlic and others), from which the garlic planting area was extracted.

Among the many classification algorithms, the RF classifier is more robust for large ranges of feature dimensionality and data noise, and the random process in the algorithm can superiorly reduce the overfitting of the model [35,36,37,38,39,40]. In addition, the RF model has become a widely used algorithm in multi-crop classification research. As a result, RF Classification [41,42,43] was used in this study. Two critical parameters determine the performance of the method. The number of decision trees is the first. Previous research has found that as the number of trees increases, the classification error or overall accuracy tends to converge. We ran tests with 50, 100, 150, and 200 decision trees. We eventually decided on 100 as the number of generated decision trees to balance calculation time and accuracy. The number of features used in the training of each decision tree is another parameter to consider. It was set to the square root of the number of input features, as recommended in the literature [44,45,46,47].

The accuracy of the crop area extraction and mapping results is mainly verified from two aspects: crop identification accuracy (i.e., location accuracy) and crop area estimation accuracy (i.e., total area accuracy) to evaluate the accuracy of the spatial distribution extraction results of garlic and the degree of consistency with the statistical data on crop planting area [48]. Crop identification accuracy was primarily used to assess the accuracy of crop spatial distribution extraction results, and the indicators primarily included the overall accuracy (OA), kappa coefficient (Kappa), producer accuracy (PA), and user accuracy (UA), all of which were obtained from the confusion matrix [14,49]. Both the OA and kappa values are between 0 and 1, and the closer the value is to 1, the more accurate the crop distribution extraction. PA and UA represent a single class’s classification accuracy. The specific calculation formula is as follows:

(1) $$\mathrm{OA}=\frac{\sum_{i=1}^{n}X_{ii}}{N^{2}}\times 100\%$$

(2) $$\mathrm{Kappa}=\frac{N\sum_{i=1}^{n}X_{ii}-\sum_{i=1}^{n}\left(X_{i+}X_{+i}\right)}{N^{2}-\sum_{i=1}^{n}\left(X_{i+}X_{+i}\right)}$$

(3) $$\mathrm{PA}=\frac{X_{ii}}{X_{+i}}\times 100\%$$

(4) $$\mathrm{UA}=\frac{X_{ii}}{X_{i+}}\times 100\%$$

where *X_ii_* refers to the number of class *i* pixels that were correctly classified, *X_i+_* denotes the number of class *i* pixels in the classification result, *X_+i_* stands for the number of class *i* pixels in the reference data, *N* is the total number of all the pixels, and *n* is the number of classes.

Furthermore, when compared to crop area statistical data, crop area estimation accuracy was mainly used to assess total area accuracy (TA). The following is how the specific total area accuracy was calculated:

(5) $$\mathrm{TA}=1-\;\frac{{|\;A}_{2}{\;-\;A}_{1\;}|}{A_{1}}\times 100\%$$

where TA is the total crop are accuracy (%); *A*_2_ is the area of garlic extracted by remote sensing (m^2^); *A*_1_ is the statistical data on the garlic planting area in the study area (m^2^).

## 4. Result and Analysis

### 4.1. Mono-Temporal Extraction

**Figure 3:**
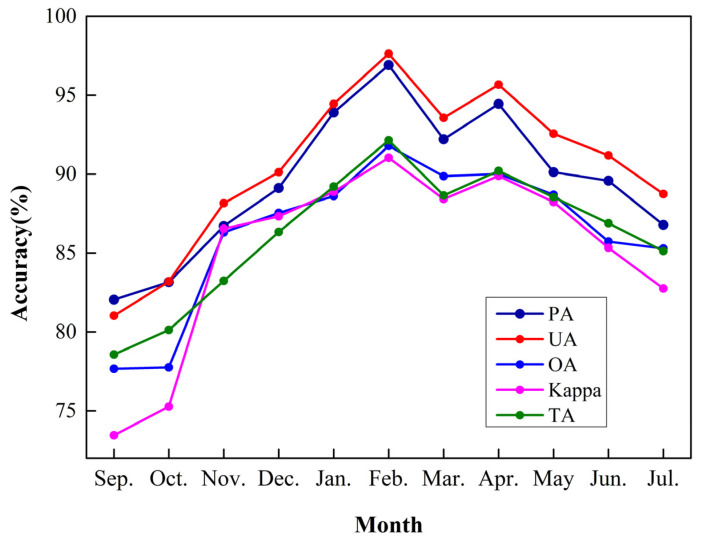
Results of accuracy indicators for each month.

Figure 3 shows five indicators (PA, UA, OA, Kappa, and TA) for garlic extraction by classifying the images of each month and depicts how the accuracy of garlic extraction varies greatly from different months. The accuracy gradually increases with time, and it grows the fastest from October to November. It could be since garlic is in the seedling stage and is tender and green. In contrast, other crops (especially for winter wheat) only have the light-yellow seedlings differ from the spectral characteristics of garlic. The accuracy peaks in February and April and then decreases gradually. In particular, the accuracy in February is the highest (OA = 91.79%, Kappa = 91.04%, and TA = 92.14%); the accuracy in September is the lowest (OA = 82.04%, Kappa = 91.04%, and TA = 78.56%).

Furthermore, the PA and UA in February are 96.91% and 97.63%, respectively, significantly higher than in previous months. Therefore, it is determined that the best month to extract garlic was February. This is because garlic sprouts slower than wheat, and garlic leaves are lighter in color and have spectral characteristics that vary from winter wheat. On the other hand, the worst result was in September, possibly because garlic and winter wheat have only recently begun to be planted, and the spectral characteristics are not significantly different.

**Figure 4:**
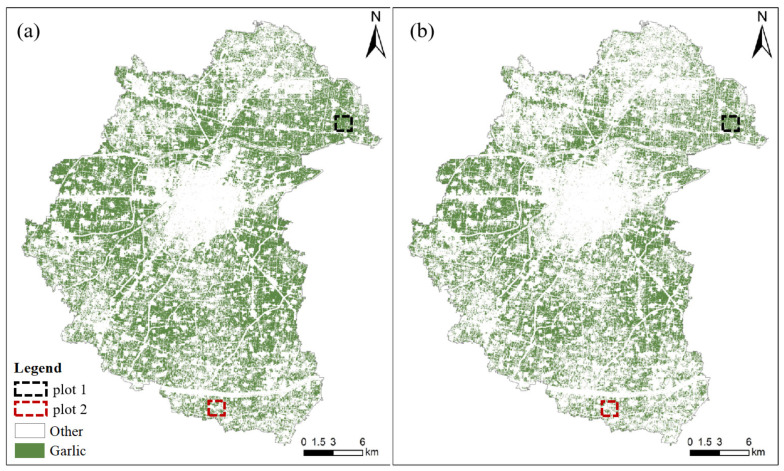
Garlic extraction results in (**a**) February and (**b**) September.

**Figure 5:**
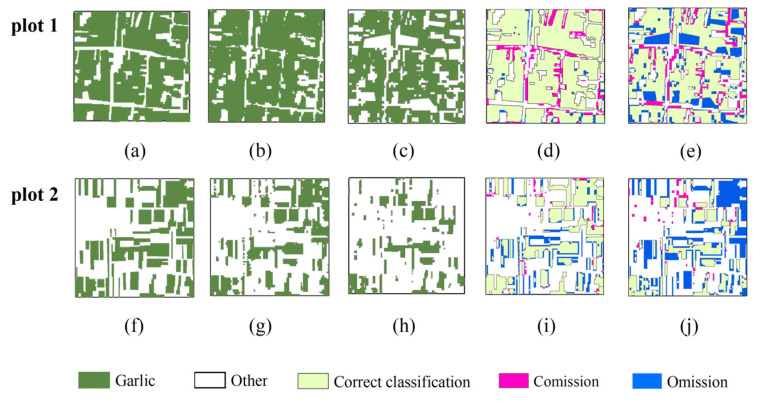
Results of accuracy validation based on two validation quadrats with an area of 1 km by 1 km. (**a**,**f**) The ground truth quadrats. (**b**,**g**) The garlic extraction results in February. (**c**,**h**) The garlic extraction results in September. (**d**,**i**) Accuracy validation results in February. (**e**,**j**) Accuracy validation results in September.

Figure 4 depicts garlic extraction results in February and September. It can be seen that the garlic extracted in September is significantly less than that extracted in February, but the overall visual effect is not apparent. Therefore, we chose two quadrats, with an area of 1 km by 1 km, better to understand the spatial distribution of correct and error results, as shown in Figure 5.

Figure 5 depicts the results of garlic extraction and accuracy verification in plot 1 (dense planting area) and plot 2 (sparse planting area). In plot 1, the commission and omission error are 8.33% and 2.6%, respectively, in February, with 13.71% and 22.83%, respectively, in September. In plot 2, the commission and omission error in February were 4.29% and 23.44%, respectively, while in September they were 8.45% and 51.3%, respectively. It can be seen that in either plot 1 or plot 2, the commission and omission error are lower in February (overwintering period) than in September (sowing period), and the garlic extraction results in February are closer to the actual distribution of garlic. In the meantime, the garlic extraction area in September is relatively fragmented, and the commission and omission are worse, and it is primarily distributed on the boundary, which is caused by spectral interference from weeds, roads, or buildings in the mixed pixels.

### 4.2. Multi-Temporal Extraction

**Figure 6:**
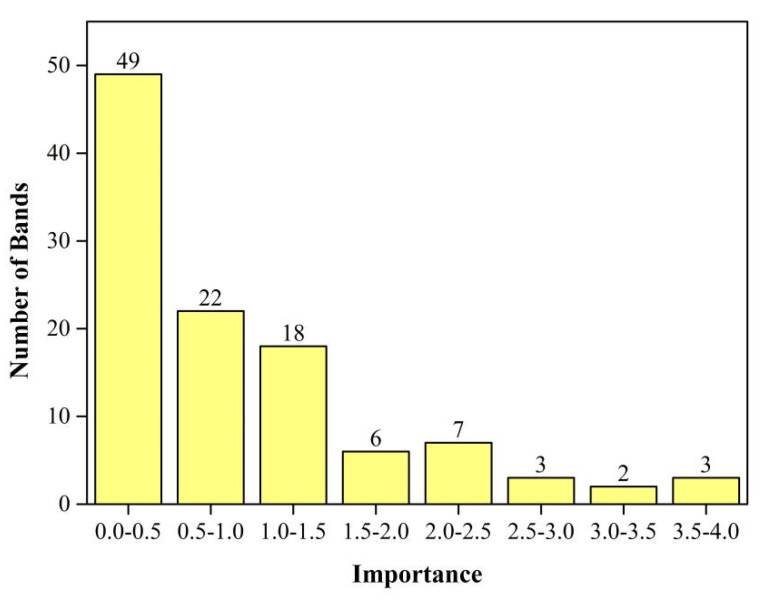
The number of bands for each scored segment.

In order to evaluate the importance of different bands, we divided the importance scores by taking into account the running time and work efficiency. In this study, we used 0.5 as the step length and divided 110 bands into eight groups. The number of bands for each score segment is depicted in Figure 6. It shows that the number of importance scores in the range of 0.0–0.5 is the greatest, with 49. The score is then in the range of 0.5–1.0, with a total of 22 bands. Thus, the scores are 2.5–3.0, 3.0–3.5, and 3.5–4.0, with three bands, two bands, and three bands, respectively.

Different schemes are designed by gradually accumulating bands of each score segment. As shown in Table 3, eight different kinds of multi-temporal schemes were designed as follows: Scheme I: choose the top 3 bands in importance, involving two months (February and April); Scheme II: choose the top 5 bands in importance, involving three months (February, April, and December). Scheme III: choose the top 8 bands in importance, involving four months (February, March, April, and December); Scheme IV: choose the top 15 bands in importance, involving five months (January, February, March, April, and December); Scheme V: choose the top 21 bands in importance, involving six months (January, February, March, April, September, and December); Scheme VI: choose the top 39 bands in importance, involving all months except July and October; Scheme VII: choose the top 61 bands in importance, involving all months except October; Scheme VIII: all 110 bands were selected, involving all months.

**Figure 7:**
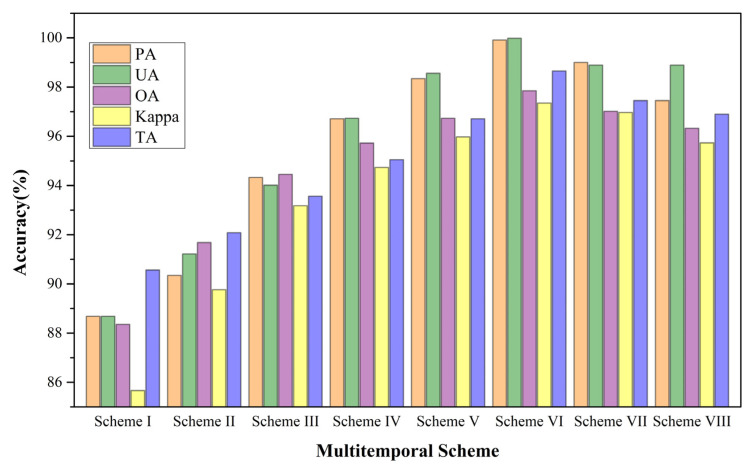
Results of accuracy indicators for each multi-temporal scheme.

Figure 7 depicts five accuracy indicators of garlic extraction (PA, UA, OA, Kappa, and TA) for eight multi-temporal schemes. As illustrated in Figure 7, as the number of bands participating increases, accuracy increases first and then decreases. From Scheme I to Scheme VI, the accuracy gradually improves. Scheme VI has the highest OA and Kappa, at 97.01 and 96.96%, respectively, and TA is 98.65%, indicating that the top 39 bands play a crucial role in garlic extraction, avoiding the interference of redundant information. Compared to Scheme I, the OA of Scheme VI has increased by 9.50%, and the Kappa has risen by 11.69%. However, the accuracy of Schemes VII and VIII fell slightly. It could be due to information redundancy, which reduces accuracy when almost all bands are added. Scheme VII has 22 bands with scores ranging from 0.5 to 1.0 (Figure 6), while Scheme VIII has 22 bands with scores ranging from 0.5 to 1.0 and 49 bands with scores ranging from 0.0 to 0.5 (Figure 6), and the importance of these bands in garlic extraction is not significant.

Furthermore, Figure 7 shows that a slight difference in accuracy between Scheme VI and Scheme III, Scheme IV, and Scheme V. Compared with Scheme VI (OA = 97.85% and Kappa = 97.35%), the OA of Scheme III was 94.45%, and the Kappa was 94.73%, and the number of bands is 8, 25 fewer than the number of bands in Scheme VI. As a result, in terms of accuracy and efficiency, Scheme III can be used instead of Scheme VI to some extent. However, because the goal of this study is to find the best multi-temporal bands combination for garlic extraction, Scheme VI has higher accuracy than Scheme III. At the same time, Scheme VI has the highest accuracy of all schemes, so Scheme VI was chosen as the best multi-temporal band combination scheme for the following research and analysis.

**Figure 8:**
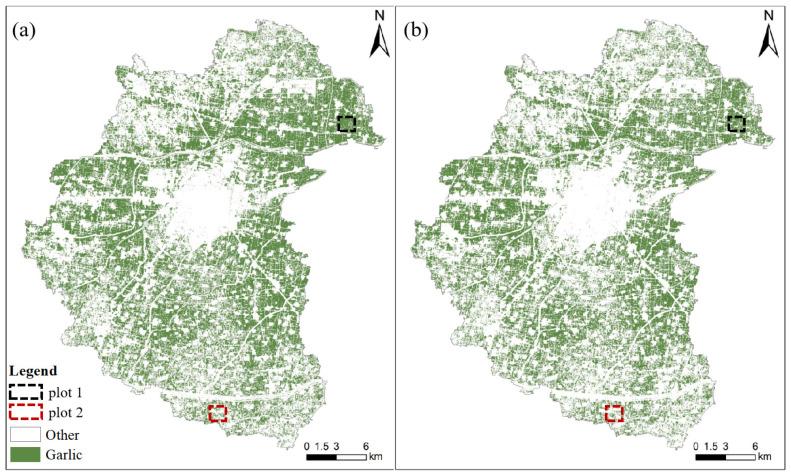
Garlic extraction results with (**a**) Scheme VI and (**b**) Scheme I.

Figure 8 depicts the garlic extraction results for Scheme VI and Scheme I. Similarly, the overall visual effect is not apparent. Therefore, we used the enlarged local map of two plots to show the two schemes’ garlic extraction performances.

**Figure 9:**
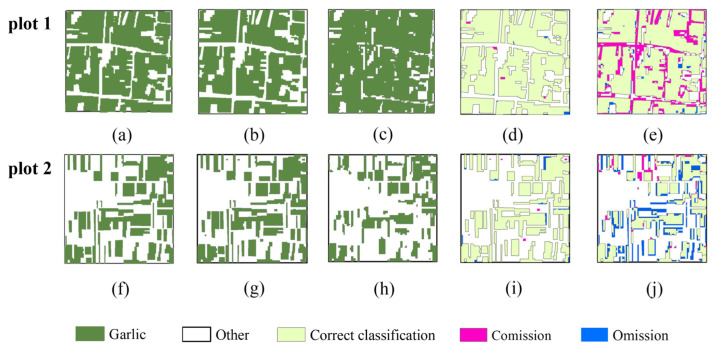
Results of accuracy validation based on two validation quadrats with an area of 1 km by 1 km. (**a**,**f**) The ground truth quadrats. (**b**,**g**) The extraction results in Scheme VI. (**c**,**h**) The garlic extraction results in Scheme I. (**d**,**i**) Accuracy validation results in Scheme VI. (**e**,**j**) Accuracy validation results in Scheme I.

Figure 9 depicts enlarged results of Scheme VI and Scheme I for plot 1 and plot 2. It can be seen that the area of garlic extracted in Scheme VI (Figure 9b,g) is complete and more suitable for the actual garlic distribution (Figure 9a,f), and the commission error is less than 1%, and the omission error is less than 3%. In addition, it is shown that the effect of garlic extraction is poor in Scheme I, with a commission error of 22.87% in plot 1 and 30.73% in plot 2. Therefore, we can conclude that Scheme VI is the best multi-temporal scheme.

**Figure 10:**
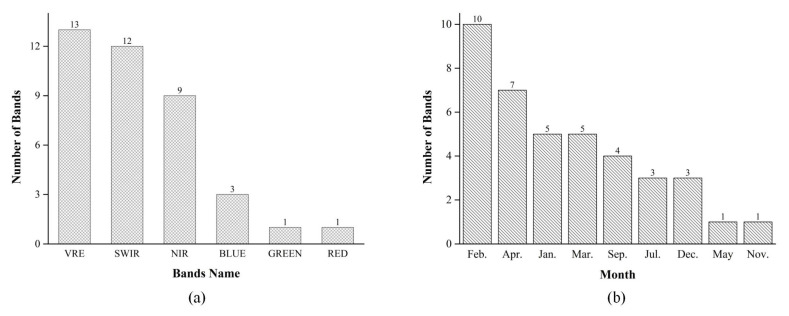
(**a**) The number of different bands in Scheme VI. (**b**) The number of different months in Scheme VI.

Further analysis of the bands of the best multi-temporal Scheme VI (the top 39 bands in importance score) found that there are differences in the proportions of different types of bands and different months when participating in garlic extraction. To more intuitively show this difference, Figure 10 shows the number of different bands and months in the best multi-temporal Scheme VI. Figure 10a shows that the number of red-edge bands is the greatest, and there are 13 red-edge bands among the 39 bands, followed by 12 shortwave-infrared bands and fewer red, green, and blue bands. It shows that the red-edge and shortwave-infrared bands play a more significant role in garlic extraction, accounting for 33.3% and 30.8%, respectively, while the red, green, and blue bands are not prominent.

Figure 10b depicts the variation in the number of months in Scheme VI. It can be seen that all 10 bands of February are in Scheme VI’s 39 bands, accounting for 25.6%, indicating that for garlic, the contribution of February to garlic extraction is the highest, which is similar to the mono-temporal result. In addition, the seven bands of April are among the 39 bands in Scheme VI. The number of bands in April account for a more significant proportion (17.9%). This is because garlic is in the bolting stage and has a blue-green color, whereas winter wheat are in the booting stage and have a dark black or dark green color, which is easy to distinguish.

### 4.3. Comparison of the Optimal Mono-Temporal and Multi-Temporal Extraction

To further explore the potential of multi-temporal bands combination for garlic extraction, the results of optimal multi-temporal (Scheme VI) and optimal mono-temporal (February) are compared. Table 4 shows the five accuracy indicators of the optimal mono-temporal and multi-temporal schemes. It is shown that the accuracy in the multi-temporal scheme is significantly improved compared to the mono-temporal scheme, with OA, kappa, and TA increasing by about 6%. It could be because the multi-temporal scheme combines influential bands from different months of the garlic growth period and fully utilizes spectral information.

**Figure 11:**
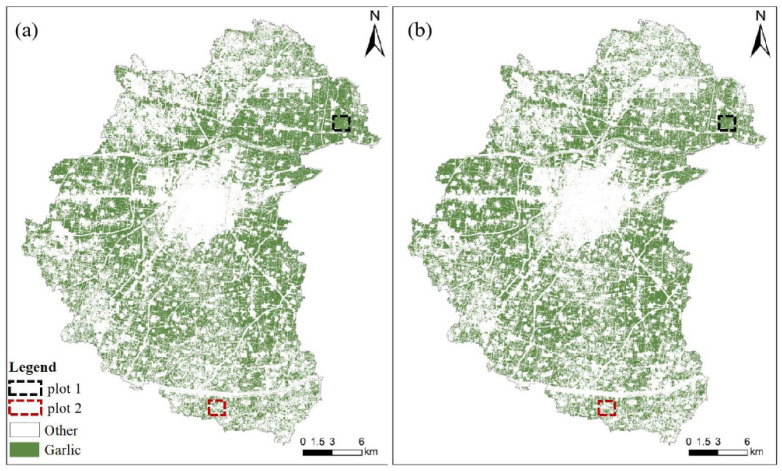
Garlic extraction results of (**a**) optimal multi-temporal and (**b**) mono-temporal schemes.

Figure 11 shows multi-temporal and mono-temporal garlic extraction diagrams. The spatial distribution of garlic extraction results is visible. The garlic planting area is concentrated in the northeast and western regions. The central part is cities and towns, with less cultivated land and less garlic planting area. The planting structure of crops is complex in the southeastern and northwestern regions, and garlic is not the main crop, so the garlic planting area is small and scattered. Similarly, the overall visual garlic extraction effect of the two schemes is unclear. Therefore, the local regions (plot 1 and plot 2) are enlarged to evaluate further the garlic extraction effect of the multi-temporal and mono-temporal schemes.

**Figure 12:**
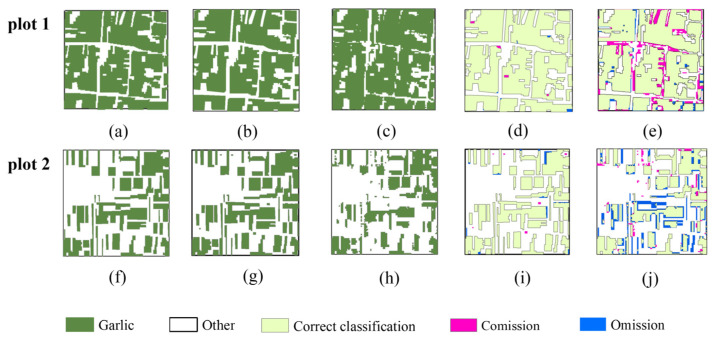
Results of accuracy validation based on two validation quadrats with an area of 1 km by 1 km. (**a**,**f**) The ground truth quadrats. (**b**,**g**) The extraction results in optimal multi-temporal scheme. (**c**,**h**) The garlic extraction results in optimal mono-temporal scheme. (**d**,**i**) Accuracy validation results in optimal multi-temporal scheme. (**e**,**j**) Accuracy validation results in optimal mono-temporal scheme.

Figure 12 depicts enlarged results of mono-temporal scheme and multi-temporal scheme for plot 1 and plot 2. The garlic planting area extracted using the multi-temporal scheme (Figure 12b,g) is more suitable for actual garlic distribution (Figure 12a,f), and the commission error is less than 1%, and the omission error is less than 3%. It can be seen that the effect of garlic extraction in the mono-temporal scheme is worse, commission error of plot 1 is 8.33%, while omission error of plot 2 is 23.34%. As a result, the multi-temporal scheme is the most effective for garlic extraction.

## 5. Discussion

### 5.1. Applicability of Different Schemes

If images are missing in some months or have a lot of cloud cover, we want to save data processing time and rush to make early predictions. This study discovered that a single Sentinel-2 image (February, wintering) could be used to extract the garlic planting area with high accuracy. In wintering period, the spectral characteristics of garlic and wheat are quite different. Garlic is lighter in color than wheat, making it easy to distinguish. It was in accordance with earlier studies that choosing an image from any month in the “Spring” imagery significantly impacted the accurate separation of farmland and other classes [50,51]. In the case of acquiring more Sentinel-2 images, we consider using this study’s multi-temporal Scheme III to obtain images from four months (February, March, April, and December) and extract the corresponding bands to extract garlic. This conclusion is similar to the previous study, which found that garlic can be extracted more effectively from Sentinel-2 images taken between November and April of the following year [25]. Still, slightly different was that the image of January was not included in the results of this study. One possible reason was that the image from January had a small amount of cloud cover and was not chosen by the RF method. Furthermore, if we can obtain sentinel-2 images for months other than July and October, and the data processing time is sufficient. As a result, we can select the multi-temporal Scheme VI and extract the corresponding bands to achieve a high-precision extraction of the garlic planting area.

### 5.2. Spectral Differences between Garlic and Wheat in Different Months

**Figure 13:**
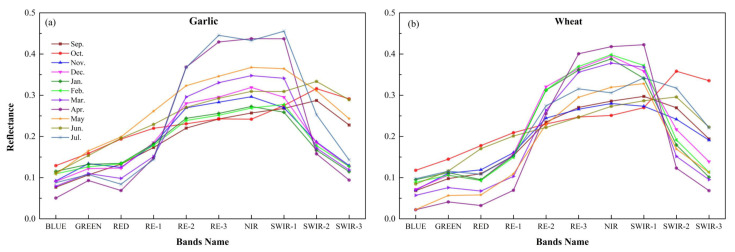
The reflectance of each month in various bands. (**a**) Garlic and (**b**) wheat.

Figure 13 depicts the difference between garlic and wheat in reflectance of each month at different bands. In Figure 13a, the reflectivity of garlic in most months fluctuates slightly in the visible band, increases in the red band, and begins to decline in the SWIR-1 band, which is consistent with the change law of the vegetation’s reflection spectrum curve. Garlic’s vegetation characteristics are especially noticeable in March and July. This could be because garlic grows more vigorously in March (reviving period). In July, because garlic was harvested, other crops were possibly grown. Vegetation characteristics of garlic are not obvious in February, January, September, and October, which could be because garlic produced small seedlings in January and February (overwintering period), which are not conducive to differentiation. In September and October, garlic was just sown, and the garlic field was entirely soil. The reflectance of wheat in Figure 13b shows noticeable vegetation characteristics from December to March of the following year. Similarly, the vegetation characteristics of wheat are not obvious in September, October, and June, which could be because the wheat was just sown in September and October and harvested in June.

It is clear that the spectra of garlic and wheat are still distinct. In particular, the vegetation characteristics of garlic are not obvious in December, and February, whereas the spectral characteristics of wheat are obvious. This could be due to the phenology differences between garlic and wheat. Garlic sprouts later than wheat. During this period, wheat grows more vigorously than garlic, which is consistent with the conclusion that February (overwintering period) is the best month for garlic extraction in this study. In addition, it can be seen that the spectral difference between garlic and wheat in April is not obvious in Figure 13, while the RF method selected April (bolting period) as the best month to distinguish garlic and wheat, which was in accordance with earlier studies that choosing the image from bolting period significantly impacted the accurate separation of wheat and other crops (garlic) [14]. It is shown that the machine learning method has good performance and can select the best month, which cannot be selected by using the difference of spectral curves between garlic and wheat. There are some limitations to selecting the best month of garlic extraction only by the difference of spectral curve. Using a machine learning method can help us mine more information conducive to garlic extraction.

### 5.3. Uncertainty and Outlook

The reference data constitute the basis for assessing the quality of the classification. Thus, these serve as verification that the research procedure performed was successful. Field investigation data have been widely used in crop extraction and mapping as reliable verification data [14,31], which will help us to assess whether the selected remote sensing data are proper for our purposes. However, because of the influence of weather and environment, this study did not conduct field investigation, so it was unable to obtain ground data, which may have resulted in insufficient accuracy verification. As a result, in the following study, we will consider using ground investigation to obtain more accurate verification data.

It should be noted that this study, we only used the spectral reflectance of the image to extract the planting area of garlic. For other crops (winter wheat, corn and soybean, etc.) whose growth cycle overlaps with garlic, we can consider using more features to extract garlic planting area, such as vegetation index, texture information, or other additional features. The vegetation index can mitigate the effects of the atmosphere, terrain, and thin clouds [52,53]. Some studies also demonstrated that spatial features such as texture information could effectively improve extraction accuracy when using mono-temporal images [54,55]. However, because this study focused on which band and which bands combination were more effective for garlic extraction, no additional features were used. It is the direction of improvement that will be considered later on. Furthermore, this study discovered that the red-edge and shortwave-infrared bands played an essential role in garlic extraction, consistent with the study findings [22,23]. Yi et al. [22] proves compared with other bands, red-edge band 1 (RE-1) and shortwave-infrared band 1 (SWIR-1) of Sentinel-2 showed a higher competence in crop classification. Liou et al. [23] proves the usefulness of shortwave infrared to increase the sensitivity of the remote sensing index on water availability and thus ability to clarify plants from the other land cover.

Previous studies have shown that, compared with traditional algorithms, machine learning models can employ data features efficiently to achieve higher classification accuracy when dealing with high dimensional and complex data spaces, such models include RF classifier, support vector machine algorithm, artificial neural network algorithm and decision tree algorithm [35,36,40,56]. However, the practice indicates that it is rare to perform object extraction with multiple machine learning methods. Therefore, it would be more beneficial to compare multiple methods on the same data (e.g., the best channel set obtained from this study) to find the most efficient method in the following study. In addition, it should be noted that this study only used the Mean Decrease Gini method in RF to rank the importance of each band because RF has the advantages of fast training speed, relatively simple implementation, and strong generalization ability [57,58]. However, because different machine learning methods use different methods to rank the importance of variables, the results may differ when using other methods to rank the importance of bands [59,60]. In the following study, we will compare the differences between different machine learning methods for the selected influential bands and find more stable bands important with multiple methods.

## 6. Conclusions

This study found that using only a few bands of multi-temporal images, rather than all bands, can achieve high-precision garlic planting area extraction. Through the comparison of eight different band combination schemes, the following conclusions can be drawn: the optimal garlic extraction scheme was multi-temporal Scheme VI (the top 39 bands in importance score), and when compared to the optimal mono-temporal scheme (February, wintering), accuracy (classification accuracy and total area accuracy) was improved by about 6%. In Scheme VI, the red-edge band and the shortwave-infrared band contributed significantly, and the bands in February and April accounted for a large proportion, indicating that the wintering and bolting stages of the garlic growing season played an essential role in garlic extraction.

The result of this study will provide important guidance for other data and crop extraction. It can assist us in determining which stages of the crop growth cycle and which bands are more critical for crop extraction. As a kind of prior knowledge, this information can reduce our demand for regular time-series data throughout the growth cycle, and it is more targeted, avoiding data redundancy and saving time. Therefore, based on this study, an automated crop extraction system can be designed to provide garlic remote sensing monitoring products for use by governments and researchers in the future. Furthermore, the follow-up will also investigate different data source combinations for crop extraction and address the issues of spectral range and spatial resolution differences between different satellites.

## Figures and Tables

**Table 1:** Summary of Sentinel-2 images used in this study.

Serial Number	Acquisition Date	Satellite Type	Growing Period	Number of Bands
1	22 September 2019	S2A	Sowing	10
2	2 October 2019	S2A	Germination	10
3	11 November 2019	S2A	Seeding	10
4	11 December 2019	S2A	Seeding	10
5	30 January 2020	S2A	Wintering	10
6	9 February 2020	S2A	Wintering	10
7	20 March 2020	S2A	Reviving	10
8	29 April 2020	S2A	Bolting	10
9	19 May 2020	S2A	Bulb expanding	10
10	3 June 2020	S2B	Harvest	10
11	8 July 2020	S2A	Harvest	10
Total				110

**Table 2:** Training and validation sample pixels for each date of images.

Class	Training Samples		Testing Samples	
	Number of Fields	Number of Pixels	Number of Fields	Number of Pixels
Garlic	97	4115	41	1618
Others	168	5055	58	2400

**Table 3:** The information of different multi-temporal schemes.

Scheme	Importance Score	Number of Bands	Bands
I	>3.5	3	RE3_2, RE2_4, RE1_2
II	>3	5	RE3_2, RE3_4, RE1_2, NIR_2, RE3_12
III	>2.5	8	RE3_2, RE3_4, RE1_2, NIR_2, RE3_12, RE1_4, SWIR2_3, RE1_3
IV	>2	15	RE3_2, RE3_4, RE1_2...RE3_3, SWIR2_2, SWIR1_3
V	>1.5	21	RE3_2, RE3_4, RE1_2...RE2_2, SWIR1_4, BLUE_1
VI	>1	39	RE3_2, RE3_4, RE1_2...NIR_7, SWIR2_11, NIR_3
VII	>0.5	61	RE3_2, RE3_4, RE1_2...RE3_7, BLUE_7, SWIR1_5
VIII	>0	110	RE3_2, RE3_4, RE1_2...GREEN_6, NIR_10, RED_10

(RE3_2 represents the red-edge band 3 in February, RE3_4 indicates the red-edge band 3 in April, RE1_ 2 indicates the red-edge band 1 in February, and so on).

**Table 4:** Accuracy of optimal multi-temporal and mono-temporal schemes.

Accuracy (%)	Optimal Multi-Temporal Scheme (Scheme VI)	Optimal Mono-Temporal Scheme (February)
PA	99.81	95.91
UA	99.98	96.63
OA	97.85	91.79
Kappa	97.35	91.04
TA	98.65	92.14

## Data Availability

The datasets presented in this study are available through: https://scihub.copernicus.eu/ (accessed on 15 January 2021), http://step.esa.int (accessed on 15 January 2021), http://www.jinxiang.gov.cn/ (accessed on 26 February 2021).
